# Pre-Existing Non-Disabling Encephalomalacia Confers Risk to Stroke Outcomes After Endovascular Treatment

**DOI:** 10.3389/fneur.2022.833737

**Published:** 2022-02-11

**Authors:** Zibao Li, Qiang Li, Yachen Ji, Zhaohu Chu, Shoucai Zhao, Lingsong Ma, Zhiming Zhou, Qian Yang, Xianjun Huang

**Affiliations:** ^1^Department of Neurology, Yijishan Hospital of Wannan Medical College, Wuhu, China; ^2^Department of Medical Record Management, Yijishan Hospital of Wannan Medical College, Wuhu, China

**Keywords:** stroke, encephalomalacia, CT, recanalization, odds ratio

## Abstract

**Background:**

Patients with previous stroke episodes tend to have poor outcomes after an endovascular treatment (EVT). Encephalomalacia (EM) is an objective indicator of previous strokes but has not been systematically investigated. The fundamental aim of this exploration is to investigate the effects of a pre-existing non-disabling EM on clinical outcomes after EVT.

**Methods:**

Consecutive patients undergoing an EVT due to the anterior circulation large vessel occlusion (LVO) strokes were enrolled in the study. The pre-existing EM was defined as the focal hypodense lesions (≥ 3 mm in maximum diameter) on a non-contrast cranial CT using axial images before EVT. The primary outcome was the 90-day functional assessment using the modified Rankin Scale (mRS) score. The safety outcome was the incidence of symptomatic intracranial hemorrhage (sICH) defined as any hemorrhage within 24 h after an EVT, which is responsible for an increase of ≥ 4 points in the score of National Institutes of Health Stroke Scale (NIHSS).

**Results:**

Of the 433 patients analyzed in this investigation, a pre-existing non-disabling EM was observed in 106 (24.5%) patients. After adjusting for potential confounding factors, patients with contralateral EM (OR = 2.68, 95% CI = 1.13–6.31; *P* = 0.025) and with an EM+ > 20 mm in maximum diameter (OR = 2.21, 95% CI = 1.01–4.85; *P* =0.048) were substantially associated with unfavorable outcomes (mRS > 2). For the sICH, we did not observe any association with the pre-existing EM (*P* > 0.05).

**Conclusions:**

A pre-existing non-disabling EM is common and safe in patients undergoing EVT. However, a contralateral EM and the large size of EM may predict an unfavorable outcome at 90 days, which should receive more attention before EVT.

## Introduction

Endovascular treatment (EVT) is commonly used as the standard of care treatment for patients with large vessel occlusion (LVO) in the anterior circulation stroke (1–7). However, the therapeutic effect of EVT varies greatly among individuals and the clinical outcomes are unpredictable, and more than half of the patients did not achieve functional independence (8). Further exploring the risk factors and optimizing the patient selection strategies are of importance in clinical practice.

Several studies have shown that the history of previous strokes may predict poor outcomes in patients with LVO who are undergoing an EVT (9–11). However, the results may be confounded by the inclusion of those patients with pre-existing disability and with different levels of baseline neurological deficits. Also, most of the stroke history data provided by the families of the patients may be inaccurate, particularly for patients without a previous disabling stroke.

Encephalomalacia (EM) on non-contrast cranial CT (NCCT) may be a better alternative to stroke history. The NCCT was used as a pretreatment screening modality for almost all the thrombectomy candidates. This approach can identify EM that occurs before the acute index stroke which could provide an objective indicator of a previous brain injury (mainly strokes). However, its relationship with clinical outcomes has not yet been investigated.

Herein, we assessed the influence of a pre-existing EM on stroke consequences after an EVT in a cohort of consecutive cases with functional independence before the index stroke. The different sides, size, and number of pre-existing EM were also analyzed.

## Methods

### Study Population

We retrospectively evaluated the information from patients with acute ischemic stroke undergoing an EVT from a prospectively collected database generated from May 2015 to August 2021. The treatment protocols for EVT have been described previously (12). The criteria for inclusion in this research were as follows: (1) age ≥ 18 years; (2) diagnosis of an acute ischemic stroke with proven proximal LVO (internal carotid artery or the M1 segment of middle cerebral artery) confirmed by digital subtraction angiography (DSA); (3) patients undergoing EVT; and (4) patients with a pre-stroke modified Rankin Scale (mRS) score ≤ 1.

The exclusion criteria for the study were as follows: (1) patients with blurring NCCT data or missing data before EVT; and (2) patients with multiple vessel occlusion (MVO) confirmed by DSA. The present survey was confirmed by the Ethics Committee of Yijishan Hospital. A written informed consent for EVT was acquired from all the patients or their guardians.

### Identification of EM on Non-Contrast Cranial CT Before EVT

A non-contrast cranial CT (NCCT) was performed on a dual-source CT scanner (SOMATOM Definition FLASH, Siemens Healthcare, Forchheim, Germany) with a slice thickness of 5 mm. The EM was defined as focal hypodense lesions (≥ 3 mm in maximum diameter) on NCCT using axial images (13, 14). We calculated the number of EM for each patient, recorded the laterality of each EM corresponding to the responsible lesion for the index stroke, and measured the maximum diameter of each EM in millimeters. In case of multiple EM, the largest maximum diameter of each patient was used for further analysis.

### Baseline Clinical and Radiologic Assessment

The demographics, medical history, and other clinical data of enrolled patients were prospectively recorded. Collateral circulation was evaluated by a retrograde contrast filling of the vessels within the occluded territory on the pretreatment DSA images. The good collaterals were defined as collateral supply filling of > 50% in the affected vascular area (15). A successful recanalization was defined as a modified Thrombolysis in Cerebral Infarction (mTICI) grade of 2b or 3 (16). The symptomatic intracranial hemorrhage (sICH) was defined as any hemorrhage confirmed by CT within 24 h that was responsible for an increase of ≥ 4 points in the score of NIHSS according to the European Cooperative Acute Stroke Study (ECASS) criteria (17). All the brain imaging data was interpreted based on the consensus of two skilled neurologists who were blinded to the clinical data of the patients and the group assignment.

### Follow-Up

The follow-up was performed by stroke neurologists through the scheduled visits or telephone interviews at 3 months following the onset of stroke. The functional outcomes were assessed using a modified Rankin Scale (mRS) score. We dichotomized the patients into favorable (mRS 0 to 2) and unfavorable outcome (mRS 3 to 6) groups.

### Statistical Analysis

The categorical variables were presented as the frequencies (percentages) and were analyzed by employing a χ2 or Fisher exact assessments as appropriate. By the mean (standard deviation, SD) or median (interquartile range, IQR), continuous variables were described and respectively analyzed using the unpaired Student *t*-tests or Mann-Whitney *U*-tests as appropriate. The analysis of logistic regression was conducted to explore the predictors of clinical outcomes. All variables with *P* < 0.1 in the analysis of univariate regression were entered into a multivariable logistic regression model in which the odds ratio (OR) with 95% CIs were evaluated. The level of statistical meaningful level was set at a two-sided *P* < 0.05. The statistical assessments were executed by implementing SPSS computer program version 23.0 (IBM Corp., Armonk, NY, USA).

## Results

**Figure 1:**
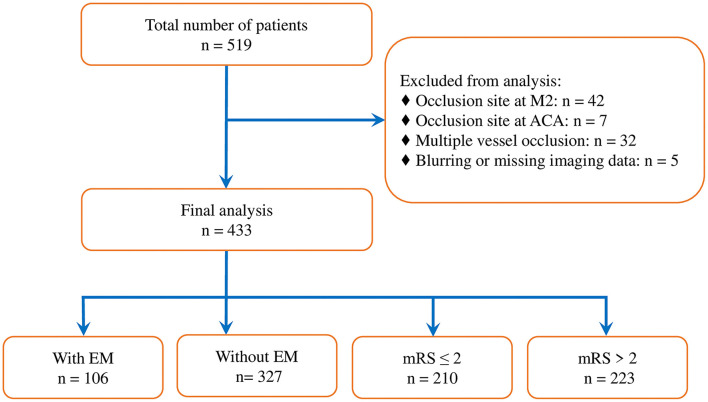
Study flowchart. M2, M2 segment of middle cerebral artery; ACA, anterior cerebral artery; EM, encephalomalacia; and mRS, modified Rankin Scale.

Of the 519 consecutive cases with anterior circulation LVO strokes processed with an EVT during the period of research, 86 cases were excluded due to the occlusion site being located at the M2 segment of the middle cerebral artery (*n* = 42), anterior cerebral artery (*n* = 7), or MVO (*n* = 32), or due to blurring or missing pretreatment imaging data (*n* = 5) (Figure 1). A total of 433 patients were enrolled in our study. Two patients who did not have postoperative CT scans for the analysis of sICH were discharged due to a sudden neurological deterioration. The baseline characteristics of studied patients are detailed in Table 1 and Supplementary Table 1.

**Table 1:** Baseline characteristics between patients with and without pre-existing encephalomalacia (EM).

**Variables**	**EM– (*n* = 327)**	**EM+ (*n* = 106)**	***P***
Demographic characteristics			
Age, y, mean (SD)	67.5 (11.1)	70.6 (10.9)	0.015
Female sex, *n* (%)	141 (43.1)	46 (43.4)	0.960
Medical history, *n* (%)			
Hypertension	209 (63.9)	77 (72.6)	0.099
Diabetes mellitus	42 (12.8)	20 (18.9)	0.124
Current smoking	91 (27.8)	35 (33.0)	0.307
Atrial fibrillation	164 (50.2)	59 (55.7)	0.324
Antithrombotics	75 (22.9)	35 (33.0)	0.038
Clinical data			
Admission SBP, mean (SD)	149 (23)	157(23)	0.004
Admission DBP, mean (SD)	83(14)	83(16)	0.614
Admission NIHSS, median, (IQR)	15 (12–18)	15 (12–19)	0.567
ASPECT score ≥ 6	303 (92.7)	96 (90.6)	0.486
IV-rtPA, *n* (%)	37 (11.3)	14 (13.2)	0.599
Occlusion site, *n* (%)			0.783
ICA	150 (45.9)	47(44.3)	
MCA-M1	177 (54.1)	59 (55.7)	
TOAST type, *n* (%)			0.193
LAA	99 (30.3)	30 (28.3)	
CE	184 (56.3)	68 (64.2)	
Others	44 (13.5)	8 (7.5)	
Procedure process			
OTP, median (IQR)	290 (223–347)	250 (219–316)	0.028
PT, median (IQR)	60 (42–90)	54 (38–81)	0.041
Good collaterals, *n* (%)	168 (51.4)	50 (47.2)	0.452
Procedural modes, *n* (%)			0.541
Solitaire FR first	192 (58.7)	68 (64.2)	
Inspiration first	90 (27.5)	27 (25.5)	
Others	45 (13.8)	11 (10.4)	
mTICI (2b/3), *n* (%)	255 (78.0)	93 (87.7)	0.028
Outcomes			
Sich	31 (9.5)	8 (7.6)	0.557
90-d mRS score ≤ 2	168 (51.4)	42 (39.6)	0.035

*EM, encephalomalacia; SBP, systolic blood pressure; DBP, diastolic blood pressure; NIHSS, National Institutes of Health Stroke Scale; SD, standard deviation; IQR, Interquartile range; ASPECTS, the Alberta Stroke Program Early Computed Tomography Score; IV-rtPA, intravenous recombinant tissue plasminogen activator; ICA, internal carotid artery; MCA, middle cerebral artery; TOAST, Trial of Org 10172 in acute stroke treatment; LAA, large artery atherosclerosis; CE, cardioembolic; OTP, onset to puncture time; PT, procedural time; mTICI, modified Thrombolysis in Cerebral Infarction; sICH, symptomatic intracranial hemorrhage; and mRS, modified Rankin Scale*.

In the study cohort, 24.5% (106/433) of the cases were assigned to the EM+ group. In comparison with cases in the EM– group, cases in the EM+ group were considerably older (70.6 ± 10.9 vs. 67.5 ± 11.1 years; *P* = 0.015), possessed a greater rate of anti-thrombotic use (33 vs. 22.9%; *P* = 0.038), higher systolic blood pressure on admission (157 ± 23 vs. 149 ± 23 mmHg; *P* = 0.004), shorter onset to puncture time (250 vs. 290 min; *P* = 0.028), shorter procedural time (54 vs. 60 min; *P* = 0.041), higher rates of successful reperfusion (87.7 vs. 78.0%; *P* = 0.028), and lower rates of favorable outcomes (39.6 vs. 51.4%; *P* = 0.035).

**Figure 2:**
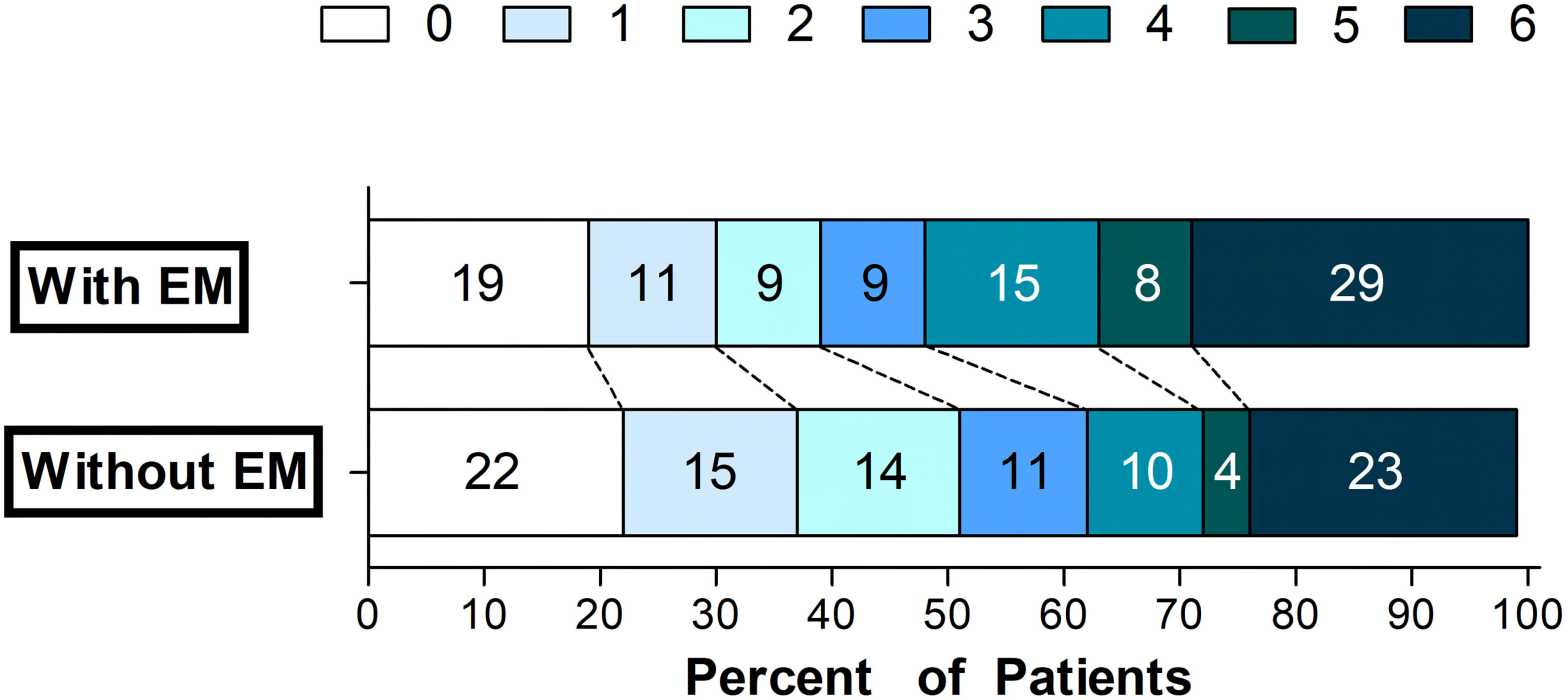
Scores on the mRS grouped by the presence of a pre-existing encephalomalacia. EM, encephalomalacia; and mRS, modified Rankin Scale.

The univariate assessment suggested that patients in the unfavorable outcome group demonstrated a greater rate of EM+ compared to those in the favorable outcome group (28.7 vs. 20%, *P* = 0.035; Table 2 and Figure 2). A contralateral EM (corresponding to the side of index event) and a larger size EM [grouped by maximum diameter of 15 mm or 20 mm which was the usual size limit for lacunes of presumed vascular origin (13, 14)] were significantly associated with unfavorable outcomes (*P* < 0.05 for all three; Table 2). However, no meaningful correlation was detected between the number of EM and stroke outcomes (Table 2). For the sICH, no association with pre-existing EM was observed (*P* > 0.05, Supplementary Table 1). After adjusting for potential confounding factors (details are provided in Supplementary Table 2), patients with contralateral EM (OR = 2.68, 95% CI = 1.13–6.31; *P* = 0.025; Table 3) or with EM+ > 20 mm in maximum diameter (OR = 2.21, 95% CI = 1.01–4.85; *P* = 0.048; Table 3) were considerably associated with unfavorable outcomes.

**Table 2:** Univariate analysis between a pre-existing encephalomalacia (EM) and a stroke outcome at 90 days.

**Variables**	**mRS ≤2 (*n* = 210)**	**mRS > 2 (*n* = 223)**	***P***
EM–	168 (80.0)	159 (71.3)	
Compared with EM–			
EM+	42 (20.0)	64 (28.7)	0.035
EM+ grouped by areas			0.011
Ipsilateral EM+	20 (9.5)	13 (5.8)	
Contralateral EM+	12 (5.7)	30 (13.5)	
EM+ on posterior circulation	3 (1.4)	6 (2.7)	
EM+ involving ≥ 2 above areas	7 (3.3)	15 (6.7)	
EM+ grouped by sizes			0.049
EM+ ≤ 15 mm in maximum diameter	19 (9.0)	21 (9.4)	
EM+ > 15 mm in maximum diameter	23 (11.0)	43 (19.3)	
EM+ grouped by sizes			0.012
EM+ ≤ 20 mm in maximum diameter	28 (13.3)	29 (13.0)	
EM+ > 20 mm in maximum diameter	14 (6.7)	35 (15.7)	
EM+ grouped by number			0.085
Number =1	31 (14.8)	43 (19.3)	
Number > 1	11 (5.2)	21 (9.4)	

*EM, encephalomalacia; mRS, modified Rankin Scale*.

**Table 3:** Multivariable logistic regression between pre-existing encephalomalacia (EM) and outcome stroke outcome at 3 months.

**Variables**	**mRS** **>** **2**		
	**Odds ratio**	**Confidence interval**	***P*-value**
Compared with EM–			
EM+	1.70	0.97–2.96	0.063
EM+ grouped by areas			0.043
Ipsilateral EM+	0.74	0.31–1.76	0.493
Contralateral EM+	2.68	1.13–6.31	0.025
EM+ on posterior circulation	4.34	0.91–20.78	0.066
EM+ involving ≥ 2 above areas	1.92	0.58–6.40	0.286
EM+ grouped by sizes			0.177
EM+ ≤ 15 mm in maximum diameter	1.64	0.71–3.76	0.244
EM+ > 15 mm in maximum diameter	1.74	0.88–3.42	0.112
EM+ grouped by sizes			0.118
EM+ ≤ 20 mm in maximum diameter	1.37	0.67–2.78	0.384
EM+ > 20 mm in maximum diameter	2.21	1.01–4.85	0.048
EM+ grouped by number			0.176
Number =1	1.73	0.92–3.27	0.091
Number > 1	1.61	0.61–4.26	0.336

*EM, encephalomalacia; mRS, modified Rankin Scale*.

*Adjusted for age, sex, hypertension, diabetes mellitus, current smoking, atrial fibrillation, antithrombotics, admission systolic blood pressure (SBP), National Institutes of Health Stroke Scale (NIHSS) score, occlusion site, Trial of Org 10172 in acute stroke treatment (TOAST), the Alberta Stroke Program Early Computed Tomography Score (ASPECTS), procedural time (PT), collateral score, procedural modes, and modified Thrombolysis in Cerebral Infarction (mTICI)*.

## Discussion

Our study demonstrated that the size and the sides of a pre-existing non-disabling EM are associated with a functional outcome, whilst no significant association was observed for the number of pre-existing non-disabling EMs. Also, the presence of pre-existing, non-disabling EM did not significantly change the incidence of sICH after the EVT.

In this study, patients in the EM+ group had initial strokes with similar severity but with worse functional outcomes compared to those in the EM– group (Table 1). The combined findings of a shorter onset to puncture time, shorter procedure times, and a higher rate of successful recanalization in the EM+ group indicate that a pre-existing non-disabling EM may impact the patient benefit from the EVT. Older age (18, 19) and higher systolic blood pressure on admission (20, 21) in the EM+ group may in part contribute to the unfavorable outcomes. Also, we hypothesize that the structural and functional changes caused by the contralateral EM, combined with the responsible focus for index stroke, may damage the bilateral brain tissues and may impair the reserve and recovery capacity. In contrast, changes caused by the ipsilateral EM might be covered by a larger focus of the index stroke. Similarly, an increased impairment of the brain reserve and recovery capacity was made in the group of EM+ (> 20 mm in maximum diameter), resulting in a relatively poor outcome after the EVT.

Previous randomized controlled studies (4–6) reported that 11–12.4% of patients with LVO undergoing an EVT had clinical documentation of previous strokes. These data were lower than the observed incidence (24.5%) of patients with a pre-existing non-disabling EM on NCCT in our study. Imaging EM in patients with minor or silent ischemia strokes is usually ignored or unrecognized during the collection of medical history and may contribute to this difference. Leker et al. (9) found that previous strokes were not associated with sICH but predicted an unfavorable outcome, which is in agreement with our findings. Kang et al. (11) reported that history of stroke/TIA was the only independent predictor of unfavorable outcomes following the EVT in cases with LVO owing to a severe intracranial atherosclerotic stenosis. These observations also supported our results.

Our results extend these findings to the patients with proximal LVO, who are undergoing an EVT, showing that a pre-existing non-disabling EM on NCCT is not a rare finding. More importantly, we provided evidence that the presence of a pre-existing non-disabling EM may confer risk to unfavorable outcomes, which may enable the further development of optimized management strategies for patients undergoing EVT. In patients with a non-disabling EM detected during the routine medical checkups, even if when there is no indication of a long-term antithrombotic use for primary prevention, an improved management of vascular risk factors and a healthy lifestyle should be advocated to prevent the occurrence of stroke. All patients and their families commonly expect favorable outcomes after stroke onset. They should be prepared to accept that patients with a pre-existing contralateral EM or a large EM may have a relatively lower rate of favorable outcomes at 90 days after EVT. If EVT is performed in these patients, a close monitoring of systolic blood pressure after admission and measures to preserve collaterals should be performed as these characteristics are important predictors for the stroke outcomes (21, 22). The prevention of hypo- and hypertension (23, 24), hypovolemia (25), hyperglycemia (24), and hyperuricemia (26) should also be considered as primary targets for intervention.

There were several restrictions that should be considered when interpreting our achievements. This study was performed retrospectively at a single-center investigation with a relatively small sample size in subgroup assessment and requires further validation in prospective multi-center studies with larger sample sizes. Due to the retrospective nature of this study, we failed to investigate the clinical relevance of a pre-existing EM according to its etiology, and the incomplete data of previous stroke history was not included in this study. The identification of a pre-existing ipsilateral EM may be disturbed in patients that have an early space-occupying effect due to a large area of cerebral infarction (7.9% patients with ASPECTS <6 in this study). The laterality of each EM, instead of the exact localization, was investigated in this study, which may affect the evaluation of neurological status. For patients with multiple pre-existing EM, the larger maximum diameter was selected for the analysis, which may have ignored the effects of the small functional lesions. However, all patients with a pre-existing EM were non-disabled in our study, which reduced the impact of the exact location and size on the evaluation of baseline neural function.

A pre-existing non-disabling EM is an objective imaging marker that is common in patients undergoing an EVT. The contralateral EM and large size of EM may predict unfavorable outcomes at 90 days and should be considered in the clinic before EVT. Larger prospective studies are warranted to validate our findings.

## Data Availability Statement

The raw data supporting the conclusions of this article will be made available by the authors, without undue reservation.

## Ethics Statement

The studies involving human participants were reviewed and approved by the Ethics Committee of Yijishan Hospital, Wuhu, China. The patients/participants provided their written informed consent to participate in this study.

## Author Contributions

ZL, QL, and YJ designed the study, analyzed all data, and prepared the manuscript. XH and QY conceptualized the study, interpreted study data, and revised the manuscript. ZZ and SZ performed interventional procedure. ZC conducted the statistical analysis. LM collected clinical data and image data. All authors approved the final manuscript.

## Funding

This study was funded by National Natural Science Foundation of China (82171329), Scientific Research Fund Project for Talent Introduction of Yijishan Hospital, Wannan Medical College in China (YR202111), and the Natural Science Research Project of Universities of Anhui Province in China (KJ2021A0843).

## Conflict of Interest

The authors declare that the research was conducted in the absence of any commercial or financial relationships that could be construed as a potential conflict of interest.

## Publisher's Note

All claims expressed in this article are solely those of the authors and do not necessarily represent those of their affiliated organizations, or those of the publisher, the editors and the reviewers. Any product that may be evaluated in this article, or claim that may be made by its manufacturer, is not guaranteed or endorsed by the publisher.
