# Supplementary material for: Pre-Existing Non-Disabling Encephalomalacia Confers Risk to Stroke Outcomes After Endovascular Treatment
Source: Front Neurol. 2022 Feb 11;13:833737. doi: 10.3389/fneur.2022.833737 (PMC8873094; doi:10.3389/fneur.2022.833737)
Supplement: Supplementary file 1 [file Table_1.DOCX]

Supplementary Material

**Supplementary Table 1** Univariate analysis between pre-existing encephalomalacia (EM) and symptomatic intracranial hemorrhage (sICH)

| **Variables** | **With sICH**  **(n=39)** | **Without sICH**  **(n=392)** | **P** |
| --- | --- | --- | --- |
| EM- | 31 (79.5) | 295 (75.3) |  |
| Compared with EM- |  |  |  |
| EM+ | 8 (20.5) | 97 (24.7) | 0.557 |
| EM+ grouped by areas |  |  | 0.567 |
| Ipsilateral EM+ | 1 (2.6) | 31 (7.9) |  |
| Contralateral EM+ | 5 (12.8) | 37 (9.4) |  |
| EM+ on posterior circulation | 1 (2.6) | 8 (2.0) |  |
| EM+ involving ≥ 2 areas above | 1 (2.6) | 21 (5.4) |  |
| EM+ grouped by sizes |  |  | 0.649 |
| EM+ ≤ 15mm in maximum diameter | 4 (10.3) | 36 (9.2) |  |
| EM+ > 15mm in maximum diameter | 4 (10.3) | 61 (15.6) |  |
| EM+ grouped by sizes |  |  | 0.810 |
| EM+ ≤ 20mm in maximum diameter | 4 (10.3) | 53 (13.5) |  |
| EM+ > 20mm in maximum diameter | 4 (10.3) | 44 (11.2) |  |
| EM+ grouped by number |  |  | 0.759 |
| Number =1 | 5 (12.8) | 68 (17.3) |  |
| Number > 1 | 3 (7.7) | 29 (7.4) |  |

EM, encephalomalacia; sICH, symptomatic intracranial hemorrhage

**Supplementary Table 2** Univariate analysis between baseline characteristics and stroke outcome at 90 days

| **Variables** | **mRS≤ 2 (n=210)** | **mRS> 2 (n=223)** | **P** |
| --- | --- | --- | --- |
| Demographic characteristics |  |  |  |
| Age, y, mean (SD) | 65.4 (10.7) | 71.0 (10.9) | < 0.001 |
| Female sex, n (%) | 74 (35.2) | 113 (50.7) | 0.001 |
| Medical History, n (%) |  |  |  |
| Hypertension | 126 (60.0) | 160 (71.7) | 0.010 |
| Diabetes mellitus | 22 (10.5) | 40 (17.9) | 0.027 |
| Current smoking | 78 (37.1) | 48 (21.5) | < 0.001 |
| Atrial fibrillation | 80 (38.1) | 143 (64.1) | < 0.001 |
| Antithrombotics | 39 (18.6) | 71 (31.8) | 0.002 |
| Clinical data |  |  |  |
| Admission SBP, mean (SD) | 148 (22) | 154(24) | 0.006 |
| Admission DBP, mean (SD) | 83(15) | 83(15) | 0.935 |
| Admission NIHSS, median, (IQR) | 13 (11-16) | 17 (13-19) | < 0.001 |
| ASPECT score ≥ 6 | 203 (96.7) | 196 (87.9) | 0.001 |
| IV-rtPA, n (%) | 25 (11.9) | 26 (11.7) | 0.937 |
| Occlusion site, n (%) |  |  | < 0.001 |
| ICA | 73 (34.8) | 124(55.6) |  |
| MCA-M1 | 137 (65.2) | 99 (44.4) |  |
| TOAST type, n (%) |  |  | < 0.001 |
| LAA | 86 (41.0) | 43 (19.3) |  |
| CE | 92 (43.8) | 160 (71.7) |  |
| Others | 32 (15.2) | 20 (9.0) |  |
| Procedure process |  |  |  |
| OTP, median (IQR) | 283 (229-341) | 270 (220-330) | 0.432 |
| PT, median (IQR) | 55 (39-76) | 70 (45-100) | < 0.001 |
| Good collaterals, n (%) | 148 (70.5) | 70 (31.4) | < 0.001 |
| Procedural modes, n (%) |  |  | 0.002 |
| Solitaire FR first | 123 (58.6) | 137 (61.4) |  |
| Inspiration first | 48 (22.9) | 69 (30.9) |  |
| Others | 39 (18.6) | 17 (7.6) |  |
| mTICI (2b/3), n (%) | 192 (91.4) | 156 (70.0) | < 0.001 |

mRS, modified Rankin Scale; SBP, systolic blood pressure; DBP, diastolic blood pressure; NIHSS, National Institutes of Health Stroke Scale; SD, standard deviation; IQR, Interquartile range; ASPECTS, the Alberta Stroke Program Early Computed Tomography Score; IV-rtPA, intravenous recombinant tissue plasminogen activator; ICA, internal carotid artery; MCA, middle cerebral artery; TOAST, Trial of Org 10172 in acute stroke treatment; LAA, large artery atherosclerosis; CE, cardioembolic; OTP, onset to puncture time; PT, procedural time; mTICI, modified Thrombolysis in Cerebral Infarction
